# Endoscopic Polipectomy with Middle Meatal Antrostomy for Antrochoanal Polyp Treatment

**DOI:** 10.1016/S1808-8694(15)30131-2

**Published:** 2015-10-19

**Authors:** Guilherme Luis da Silva Franche, Eduardo Homrich Granzotto, Andresa Thier de Borba, Fernando Hermes, Cátia de Souza Saleh, Person Antunes de Souza

**Affiliations:** a M.S. in Medicine. Head of the Rhinology ward - Santa Casa de Porto Alegre; b 3rd year resident in Otorhinolaryngology - Santa Casa de Porto Alegre; c 2nd year resident in Otorhinolaryngology - Santa Casa de Porto Alegre; d 1st year resident in Otorhinolaryngology - Santa Casa de Porto Alegre; e Medical student - Fundação Faculdade Federal de Ciências Médicas de Porto Alegre; f MD. Graduated by the Universidade Federal de Pelotas. Otorhinolaryngology Department of the Santa Casa de Porto Alegre teaching hospital

**Keywords:** endoscopic surgery, nasal polyposis, antrochoanal polyp

## Abstract

Antrochoanal polyp (ACP) or Killian polyp is a benign nonatopic lesion of the maxillary sinus. Patients usually present nasal obstruction. Many surgical options for the treatment of ACPs have been suggested to minimize postoperative recurrence. The endoscopic nasal approach is a surgical option for maxillary intrasinusal resection of the polyp implantation through the maxillary ostium or middle meatal antrostomy, with lower morbidity when compared to other surgical approaches. **Aim:** To evaluate the rate of endoscopic antrochoanal polypectomy with middle meatotomy in the treatment of ACP. **Materials and Methods:** Were evaluated by means of a retrospective study, 29 patients, who were diagnosed based on history, physical examination, computed tomography, and histological findings, treated between 1997 and 2004. The surgical approach was endoscopic polypectomy with middle meatotomy. **Results:** Twenty-nine patients with ACP, 17(58.6%) were females and 12(41.4%) males, age range, 7-75 years (average of 27.55years) were included in this study. The main symptom were nasal obstruction 24(82%), oral breathing 11(37.9%), snoring nine (31%), rhinorrhea 5(17%), epistaxis 2(6.9%), headache 2(6.9%), and drip one (3.4%). The association with atopy was found in nine (31%). The mean follow-up period was 17 months (3-63 months). Only two patients (6.9%) presented recurrence. **Conclusion:** The rate of recurrence obtained in our study is no different from literature data, even when compared with former and gold standard procedures.

## INTRODUCTION

Antrochoanal polyps (ACP), also known as Killian’s polyps, are a non-atopic^1^, benign lesions, which stem from the maxillary sinus, go through its ostium (can be the true ostium or the accessory ostium) and extends all the way to the choanas.^2^ It is more frequent in men than women, and more prevalent in children and young adults^3^, however it may manifest at any age. The distal antral origin is the most common, although ethmoid-choanal, sphenoid-choanal and choanal manifestations are occasionally seen^4^.

Presentation at physical exam is a nasal and/or rhino-pharyngeal polypoid tumoral mass, usually unilateral. Paranasal sinuses x-ray exams show a unilateral veiling of the maxillary sinus, often times with the ethmoid opaque and, depending on the size of the polypoid tumor, a mass that fills up the nasal cavity, all the way to the rhinopharynx^5^. It is clinically manifested by unilateral nasal obstruction; there may also be epistaxis, sleep disorders, postnasal drip, headaches, rhinorrhea, oral breathing and hyposmia^3^, ^5^.

Since 1906, when Killian described the maxillary sinus as the site of origin for the polyp, many surgical techniques have been proposed^5^. In order to reduce post-operative recurrence, it is paramount to completely remove the antral portion of the polyp, close to its base of origin^2^. The main techniques developed to reach this goal are the Caldwell-Luc procedure, endoscopic polypectomy with middle meatotomy, endoscopic polypectomy with antrostomy through the inferior meatus and endoscopic polypectomy with middle meatotomy and the use of microshaver with or without transcanine access.

The goal of the present investigation is to assess the rate of recurrence in endoscopic meatotomy with middle meatotomy in the treatment of ACP. As for secondary goals, we assessed symptoms, age, gender, time of follow up, association with asthma or rhinitis and post-operative complications in the patients.

## MATERIALS AND METHODS

We retrospectively assessed 29 patients diagnosed with Killian’s polyps submitted to surgery in our service in the period of 1997 to 2004, after approval by the Ethics Committee (protocol #1513/07).

In order for the patients to be included in the study, they had to be diagnosed with the antrochoanal polyp by clinical exam, radiography and anatomopathology. All the cases were operated by endoscopic polypectomy with middle meatotomy by the same surgical team.

We revised the distribution in terms of age, gender, side, symptoms, duration of follow up, atopic comorbidities, postoperative complications and recurrence rate in the population studied.

## RESULTS

We included 29 patients with antrochoanal polyps in this study, 17 (58.6%) women and 12 (41.4%) men, with ages varying between 7 and 75 years (mean age = 27.55 years). All the patients presented unilateral Killian polyps, 17 (58.6%) on the left side and 12 (41.4%) on the right.

Follow up varied between 3 and 63 months, and the mean value was 17 months. The major symptoms reported by the patients are summarized on Table 1. In assessing comorbidities, we observed 2 cases (6.9%) associated with asthma, 9 (31%) with rhinitis and 2 (6.9%) with septal deviation.

**Table 1:** Preoperative symptoms.

Symptoms	Franche G et al.	Balwant SG et al.	Hong SK et al.
Nasal obstruction	24 (83%)	17 (94%)	28 (100%)
Epistaxis	2 (7%)	6 (33%)	0
Snoring	9 (31%)	4 (22%)	-
Oral Breathing	11 (38%)	6 (33%)	-
Postnasal drip	1 (4%)	5 (28%)	-
Headache	2 (7%)	2 (11%)	-
Rhinorrhea	5 (17%)	8 (44%)	19 (67%)

Only 1 patient evolved with purulent secretion and fever in the postoperative, with a good response to clinical treatment. No patient had transoperative complications.

Recurrence rate in our study was of 6.9% (2 cases). Both patients were 8 years old and the diagnosis was made after 24 and 30 months of follow up.

## DISCUSSION

Palfyn was the first author to describe ACP among nasopharyngeal polyps in 1753; however, it was Killian in 1906, who documented the exact place of origin in the mucosa of the maxillary sinus wall.1

The antrochoanal polyp suffers the anatomical limitation of the lateral nasal wall, especially the middle meatus and the antrum, looking like a dumbbell. It usually stems from the postero-lateral wall of the maxillary sinus and it passes, without bone destruction, through the maxillary ostium, or occasionally through the accessory ostium (anterior or posterior to nasal fontanels), to inside the middle meatus. Usually the polyp takes up the space between the lateral wall conchae and grows posteriorly to reach the choana.^1^

ACPs are usually unilateral and occur in children. About 4% to 6% of nasal polyps are ACPs, and they are more common in men than in women. In our series we had a broad age range 7-75 years (mean age of 27.55 years), and despite the high mean age value, it is in accordance with other authors such as Hong SK and Gendeh BS.^2^, ^3^ All the cases were unilateral with a discreet preference to the left side. We observed a subtle female prevalence (male/female rate: 1:1.42) which is not in agreement with most authors, however Gendeh BS et al. found a ratio similar to the one we found: 1:1.5.

Asthma was seen in 2 (6.9%) of our patients and allergic rhinitis in 9 (31%). Both cases of asthma had allergic rhinitis; therefore, the total number of patients with allergy in our study was 9 (31%). Although Cook et al. found significant correlations with allergy and asthma among the 33 cases studied by the group6, most of the other papers are not associated with the cause for allergy.^7^, ^8^

Patients usually have uni or bilateral nasal obstruction. In the pediatric population, it is common to see sleep disorders and oral breathing. In adults, nighttime snoring and headaches may also be present^3^. Table 1 shows a comparison between our results, symptom-wise with those from other authors. We know that many of the symptoms can vary according to the severity found in the selected patients, as well as the climate in the region, however there was very little variation between the studies.

Simple polyp removal is associated with a 25% polypoid tumor recurrence. It is important to identify the origin of each one of these polyps, since the sinusal component must also be properly resected in order to avoid recurrences^5^.

Many surgical options to treat ACPs have been suggested in order to reduce post-operative recurrence. The ACP’s antrum portion must be completely removed. The ACP removal surgery by the classic Caldwell-Luc approach is being revised since nasosinusal endoscopic surgery^9^ became available.

Cadwell-Luc’s approach is advocated because it is very successful for the complete removal of polypoid tissue, since it offers a good exposure to remove the polyp’s antral portion. However, such technique is associated with a higher risk of injuring the infra-orbitary nerve, postoperative maxillary edema, and longer hospital stay. Such procedure is not recommended to treat children because it breaks dentition and facial growth.^3^

Endoscopy is being used as a therapeutic option in maxillary intra-sinusal resection of the polyp through the maxillary ostium, through middle meatus antrostomy, inferior meatal antrostomy or with the help of the microshaver and access through the canine fossa with less morbidity than the classic approach.

Each technique has its disadvantage described in the literature. Endoscopic polypectomy with inferior meatal antrostomy has a higher risk of the patient developing lower synechia and its efficacy is still controversial according to the literature.^3^ Endoscopic polypectomy with middle meatotomy bears satisfactory results, however access makes it difficult to remove the anterior and inferior wall insertions, and surgery time is longer when compared to other approaches.^2^ Endoscopic polypectomy with the use of a microshaver and transcanine punction is proving to be very promising according to the literature, facilitating access to the polyp’s insertion zones and reducing surgical time. However, transcanine punction may have operative complications such as the ones found in the Caldwell-Luc procedure. In our service, we use middle meatus endoscopic polypectomy.

The only surgical complication found by our team was sinusitis fifteen days after surgery, which was treated with augmented amoxicillin and the patient went symptom-free in 10 days.

Pinilla et al. also studied 12 cases of ACP treated by the endoscopic approach, assessing results and complications. Such author concluded that this approach is efficient and bears little morbidity^9^.

Rugina et al. analyzed 19 cases in a retrospective study of ACP by endoscopic middle meatotomy, reported only one relapse (5.3%), and concluded that such technique is safe and non-invasive. This author recommends the removal of the ACP’s pedicle with coagulation on the mucosal site where it was implanted^10^.

Loury et al. assessed 5 cases of Killian polyps and endoscopic surgery was an alternative approach to transantral approach. In their study, there was only one relapse (20%). Thus, the author believes that transantral endoscopic antrochoanal polypectomy is an excellent surgical option, with significantly lower postoperative morbidity when compared to the transnasal approach with similar cure indices^11^.

Schramm and Effron previously assessed 32 children, with ages between 7 and 16 years, who were submitted to a primary or secondary Caldwell-Luc procedure to remove an antrochoanal polyp. Only one of these children required surgical re-intervention because of polyp recurrence. The rate of such surgical complication was 3% for all surgical procedures, equal to what was reported in the adult literature for the Caldwell-Luc procedures^12^.

ACP’s recurrence rate in our study was 6.9% (2 cases), knowing that the diagnosis of recurrence was established at 24 and 30 months after the first surgery. We know that the average follow up time in our study (17 months), was much shorter than patients’ recurrence rate, however, the time span mentioned for recurrences started when the pathology result was established for each case. Since the suspicion of recurrence until the definitive surgery with material sent for pathology exam, some months passed; therefore, we believe our follow up time was satisfactory.

## CONCLUSION

In our service, we had a 6.9% recurrence rate, matching literature data for the treatment of ACPs. The technique utilized was endoscopic polypectomy by middle meatotomy, procedure that is not totally established because of the lack of well-outlined studies comparing it with the standard approach. Our study reinforces the efficacy and low morbidity associated with the procedure at hand.
